# Construction and validation of a nomogram model for lymph node metastasis of stage II-III gastric cancer based on machine learning algorithms

**DOI:** 10.3389/fonc.2024.1399970

**Published:** 2024-10-08

**Authors:** Chongkang Yue, Huiping Xue

**Affiliations:** Department of Gastroenterology and Hepatology, Shanghai Institute of Digestive Disease, Renji Hospital, Shanghai Jiao Tong University School of Medicine, Shanghai, China

**Keywords:** gastric cancer, lymph node metastasis, machine learning, nomogram, prediction model

## Abstract

**Background:**

Gastric cancer, a pervasive malignancy globally, often presents with regional lymph node metastasis (LNM), profoundly impacting prognosis and treatment options. Existing clinical methods for determining the presence of LNM are not precise enough, necessitating the development of an accurate risk prediction model.

**Objective:**

Our primary objective was to employ machine learning algorithms to identify risk factors for LNM and establish a precise prediction model for stage II-III gastric cancer.

**Methods:**

A study was conducted at Renji Hospital Affiliated to Shanghai Jiao Tong University School of Medicine between May 2010 and December 2022. This retrospective study analyzed 1147 surgeries for gastric cancer and explored the clinicopathological differences between LNM and non-LNM cohorts. Utilizing univariate logistic regression and two machine learning methodologies—Least absolute shrinkage and selection operator (LASSO) and random forest (RF)—we identified vascular invasion, maximum tumor diameter, percentage of monocytes, hematocrit (HCT), and lymphocyte-monocyte ratio (LMR) as salient factors and consolidated them into a nomogram model. The area under the receiver operating characteristic (ROC) curve (AUC), calibration curves, and decision curves were used to evaluate the test efficacy of the nomogram. Shapley Additive Explanation (SHAP) values were utilized to illustrate the predictive impact of each feature on the model’s output.

**Results:**

Significant differences in tumor characteristics were discerned between LNM and non-LNM cohorts through appropriate statistical methods. A nomogram, incorporating vascular invasion, maximum tumor diameter, percentage of monocytes, HCT, and LMR, was developed and exhibited satisfactory predictive capabilities with an AUC of 0.787 (95% CI: 0.749-0.824) in the training set and 0.753 (95% CI: 0.694-0.812) in the validation set. Calibration curves and decision curves affirmed the nomogram’s predictive accuracy.

**Conclusion:**

In conclusion, leveraging machine learning algorithms, we devised a nomogram for precise LNM risk prognostication in stage II-III gastric cancer, offering a valuable tool for tailored risk assessment in clinical decision-making.

## Introduction

Gastric cancer, a pervasive malignancy within the gastrointestinal tract, stands as the fifth most prevalent global malignant tumor and constitutes the third leading cause of cancer-related mortality worldwide (1). According to statistics, more than 1 million people are diagnosed with gastric cancer annually. Unfortunately, the 5-year survival rate of gastric cancer scarcely breaches the 20% threshold globally (2). The incidence of gastric cancer exhibits discernible regional predilections, with a notable surge in incidence observed in East Asia and Eastern Europe, in stark contrast to the relatively diminished rates witnessed in Northern Europe and North America (3). The lack of obvious clinical symptoms in early gastric cancer engenders a formidable hurdle in the realms of both effective diagnosis and intervention (4). Once diagnosed with advanced gastric cancer, about 80% of these patients have regional LNM. The presence or absence of LNM affects the prognosis and treatment options of patients (5, 6). Regrettably, current clinical methodologies, exemplified by gastroscopy and abdominal-enhanced CT scans, languish in poor accuracy when detecting LNM. Hence, it is particularly crucial to develop a risk prediction model and meticulously evaluate the looming risk of LNM in gastric cancer patients before surgical intervention.

In recent years, the rapid evolution of diverse machine learning algorithms has burgeoned, finding expansive applications in the medical domain to discern intricate patterns and relationships within complex clinical parameters, thus facilitating precise decision-making (7, 8). RF and LASSO stand out as prominent machine learning algorithms because of their capacity to simulate and predict intricate relationships between variables and outcomes. Wu et al. used the LASSO algorithm to establish the nomogram model of early gastric cancer LNM (9). Tian et al. employed seven machine learning algorithms including LASSO and RF to prognosticate the risk of LNM in early gastric cancer across diverse ethnic cohorts (10). To date, existing literature rarely explores machine learning model in predicting II-III stage gastric cancer risk of LNM.

In this study, we undertook a comprehensive approach, employing univariate logistic regression and two machine learning methods: LASSO and RF algorithms to sift through potential risk factors contributing to LNM in gastric cancer of II-III stage. The convergence of identified risk factors across all three analytical methods served as the basis for establishing a nomogram model within the training set, subsequently subjecting it to validation in an internal validation set. ROC curves, calibration curves, and decision curves were carried out to evaluate the predictive efficacy of the nomogram.

## Materials and methods

### Subjects

**Figure 1:**
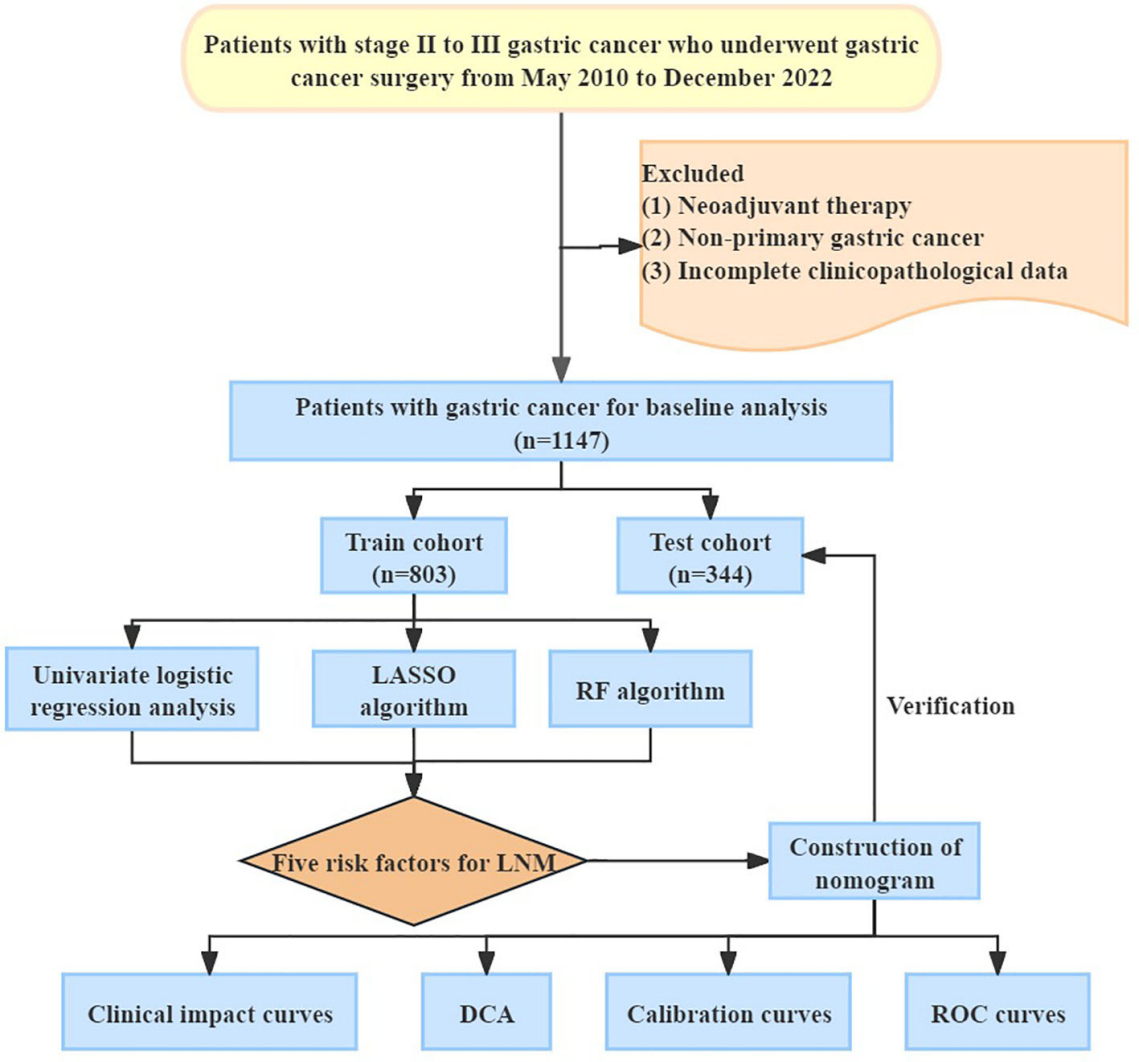
Overview procedure of the research. LASSO, Least Absolute Shrinkage and Selection Operator; RF, Random Forest; LNM, Lymph Node Metastasis; DCA, Decision Curve Analysis; ROC, Receiver Operating Characteristic Curve.

**Figure 1** showed the procedure of our research. The investigation received ethical clearance from the Ethics Committee of Renji Hospital Affiliated to Shanghai Jiaotong University School of Medicine (Approval letter number: LY2023-273-B). A retrospective analysis encompassing a cohort of 1147 patients diagnosed with gastric cancer, who underwent surgical interventions at this institution from May 2010 to December 2022. The types of surgery performed comprised radical gastrectomy, total gastrectomy, and palliative gastrectomy. The inclusion and exclusion criteria were as follows. Inclusion criteria: (1) Patients with stage II-III gastric cancer diagnosed by surgery and postoperative pathological assessments adhering to the 8th edition of the American Joint Committee on Cancer (AJCC) staging system (11). (2) Comprehensive clinicopathological data. Exclusion criteria: (1) Patients with stage I or IV gastric cancer diagnosed by AJCC staging system; (2) Patients receiving neoadjuvant therapy before surgery; (3) Patients with malignant tumors originating in other anatomical sites but exhibiting gastric metastasis.

### Data collection and processing

Patient demographic data, including age and gender, along with preoperative peripheral blood indicators, such as peripheral blood cell counts, liver and renal function tests, lymphocyte-monocyte ratio (LMR), neutrophil-lymphocyte ratio (NLR) (12), platelet-lymphocyte ratio (PLR) (13), prognostic nutritional index (PNI) (14), and systemic immune-inflammation index (SII) (15), were systematically gathered. Based on existing studies, the PNI can be calculated as the sum of albumin levels (g/L) and five times the lymphocyte count (10^9/L). Similarly, the SII was determined by multiplying platelet count with neutrophil count and dividing it by lymphocyte count (16). Tumor pathology parameters including tumor location, maximum diameter, nerve invasion, vascular invasion, esophageal invasion, differentiation type, gross morphology, depth of invasion, lymph node metastasis, and microscopic identification of signet ring cells, were comprehensively documented. Additionally, the study encompassed the duration of hospital stay and details regarding the employed surgical methods. To assist statistical analysis, a classification system was used to differentiate between high, medium, and low tumor differentiation, as well as other cancer types like signet ring cell carcinoma and mucinous adenocarcinoma.

### Machine learning algorithms to screen the risk factors for LNM of gastric cancer

Utilizing the caret and randomForest packages within the R software, we employed machine learning algorithms, specifically the LASSO and RF, to meticulously scrutinize the risk factors associated with LNM in gastric cancer. LASSO, a regression-based machine learning technique, emerged as a pivotal tool for feature selection and regularization, facilitating the identification of a pertinent subset of predictor variables essential for predicting the outcomes of interest. Its intrinsic ability to navigate feature relevance mitigates the peril of overfitting, ensuring the model’s robustness (17, 18). Concurrently, the RF algorithm, an ensemble learning method, was harnessed to amalgamate insights from multiple decision trees, thereby enhancing the precision of predictions. Its versatility extends to handling both categorical and continuous data, and its inherent robustness effectively guards against overfitting, a crucial consideration in complex datasets (19).

### Establishment and validation of a nomogram

Employing the CreateDataPartition function within the R software, we randomly allocated 1147 gastric cancer patients into training and validation sets at a ratio of 7:3. In the training set, the “rms” R package was utilized to craft the nomogram. Each predictor had a corresponding score, and the total score represented the sum of the scores of the above predictors. Subsequently, ROC curves, facilitated by the “pROC” R package, were undertaken to gauge the predictive efficacy of LNM factors within both the training and validation sets. The AUC values served as a robust metric for this assessment. Calibration curves and decision curve analysis (DCA) were further leveraged to appraise the nomogram model’s predictive accuracy. Shapley Additive Explanation (SHAP) values were employed to measure the individual contributions of each feature in the model.

### Statistical methods

All statistical analyses were carried out using R software (version: 4.20) and SPSS software (version 26.0). Categorical variables were expressed as cases (%) and subjected to scrutiny via the chi-square test to ascertain statistical differences. To compare the two groups, we used either the Wilcoxon rank-sum test or the Student’s t-test, depending on the data’s distribution and assumptions. For more than two groups, we applied one-way ANOVA as a parametric method and the Kruskal-Wallis test as a nonparametric method. All statistical tests were inherently two-sided, with statistical significance conventionally set at p-values < 0.05.

## Results

### Clinical characteristics of study subjects

**Table 1** meticulously displayed the clinical characteristics and the occurrences of LNM among 1147 patients with stage II-III gastric cancer. Within this cohort, there were 806 male patients (70.3%) and 341 female patients (29.7%). The median age of all patients was 65 years. The median duration of hospitalization was observed as 16 days. A total of 385 individuals (33.6%) manifested stage II gastric cancer, while 762 (66.4%) confronted the more advanced stage III. A total of 869 patients (83%) exhibited LNM, while 278 patients (17%) did not exhibit lymph node metastasis. In terms of tumor-specific data after surgery, 470 cases were found in the antrum (41%). Among all cases, Borrmann type 3 gastric cancer accounted for 666 instances (58.1%), and poorly differentiated tumors were detected in 738 patients (64.3%). Nerve invasion occurred in 45.9% of patients, vascular invasion in 47.5%, and esophageal invasion only manifested in a mere 9.9% of cases. As shown in **Table 1** and **Supplementary Table 1**, statistical analyses unveiled significant associations (p < 0.05) between LNM and pivotal factors, including tumor location, maximum tumor diameter, differentiation type, Borrmann type, depth of tumor invasion, neural invasion, vessel invasion, length of hospital stay, absolute monocyte count, HCT, mean corpuscular volume (MCV), mean corpuscular hemoglobin (MCH), platelet (PLT), total protein, albumin, albumin/globulin (A/G), pre-albumin, PLR, LMR, SII, and PNI. Conversely, parameters such as gender, age, microscopic signet ring cells, and other peripheral blood indicators did not exhibit a discernible association with LNM.For further research, 1147 stage II-III gastric cancer patients were randomized at a ratio of 7:3, of which 803 were assigned to the training set and 344 to the validation set. As elucidated in **Supplementary Table 2**, a comprehensive scrutiny of clinical-pathological features revealed no statistically significant differences, thereby ensuring the homogeneity of both the training and validation sets (p > 0.05).

**Table 1:** Clinical features and lymph node metastasis of 1147 patients with gastric cancer.

Covariates	Type	Total	N0	N1-3	P value
Sex	Female	341 (29.7%)	89 (32%)	252 (29%)	0.338
	Male	806 (70.3%)	189 (68%)	617 (71%)	
Age (yr)		65.00 (57.00-73.00)	66.00 (57.00-73.00)	65.00 (57.00-73.00)	0.716
Tumor location	Autrum	470 (41.0%)	117 (42.1%)	353 (40.6%)	0.003
	Body	398 (34.7%)	104 (37.4%)	294 (33.8%)	
	Cardia	234 (20.4%)	55 (19.8%)	179 (20.6%)	
	Whole	45 (3.9%)	2 (0.7%)	43 (4.9%)	
Tumor size (cm)		5.00 (3.00-7.00)	3.50 (4.00-7.00)	5.00 (4.00-7.00)	<0.0001
Differentiation type	Well	22 (1.9%)	9 (3.2%)	13 (1.5%)	0.001
	Moderate	251 (21.9%)	81 (29.1%)	170 (19.6%)	
	Poor	738 (64.3%)	158 (56.8%)	580 (66.7%)	
	Other type	136 (11.9%)	30 (10.8%)	106 (12.2%)	
TNM stage	II	385 (33.6%)	262 (94.2%)	123 (14.2%)	<0.0001
	III	762 (66.4%)	16 (5.8%)	746 (85.8%)	
Borrmann type	1	90 (7.8%)	48 (17.3%)	42 (4.8%)	<0.0001
	2	148 (12.9%)	40 (14.4%)	108 (12.4%)	
	3	666 (58.1%)	147 (52.9%)	519 (59.7%)	
	4	243 (21.2%)	43 (15.5%)	200 (23%)	
T	T1	49 (4.3%)	29 (10.4%)	20 (2.3%)	<0.0001
	T2	107 (9.3%)	35 (12.6%)	72 (8.3%)	
	T3	113 (9.9%)	27 (9.7%)	86 (9.9%)	
	T4	878 (76.5%)	187 (67.3%)	691 (79.5%)	
Neural invasion	No	621 (54.1%)	173 (62.2%)	448 (51.6%)	0.002
	Yes	526 (45.9%)	105 (37.8%)	421 (48.4%)	
Vessel invasion	No	602 (52.5%)	217 (78.1%)	385 (44.3%)	<0.0001
	Yes	545 (47.5%)	61 (21.9%)	484 (55.7%)	
Esophageal invasion	No	1034 (90.1%)	253 (91%)	781 (89.9%)	0.581
	Yes	113 (9.9%)	12 (9%)	88 (10.1%)	
Signet ring cell	No	869 (75.8%)	218 (78.4%)	651 (74.9%)	0.235
	Yes	278 (24.2%)	60 (21.6%)	218 (25.1%)	
Hospitalization (d)		16.00 (12.96-21.00)	15.00 (13.00-21.00)	16.00 (13.00-21.00)	0.005

### Identification of LNM risk factors

Initially, univariate logistic regression scrutinized potential risk factors for LNM in gastric cancer, revealing that tumor location (OR=8.75, P=0.036), Borrmann type (OR=4.65, P<0.001), maximum tumor diameter (OR=1.34, P<0.001), depth of tumor invasion (OR=6.07, P<0.001), vascular invasion (OR=4.25, P<0.001), length of hospital stay (OR=1.02, P=0.029), absolute monocyte count (OR=2.72, P=0.02), HCT (OR=0.98, P<0.001), LMR (OR=0.92, P=0.017), PNI (OR=0.98, P=0.049), albumin (OR=0.96, P=0.015), A/G (OR=0.43, P=0.004) were significant contributors (**Table 2**). Employing a significance threshold of P < 0.1, additional factors, namely neural invasion, percentage of lymphocytes, percentage of monocytes, MCV, and pre-albumin were earmarked for subsequent analysis.

**Table 2:** Univariate analysis of risk factors for LNM in patients with gastric cancer.

Covariates	Type	OR (Univariable)
Tumor location	Cardia	
	Body	0.76 (0.48-1.20, p=0.234)
	Autrum	0.94 (0.60-1.48, p=0.779)
	Whole	8.75 (1.16-66.13, p=0.036)
Borrmann type	1	
	2	2.63 (1.37-5.06, p=0.004)
	3	3.37 (1.96-5.79, p<0.001)
	4	4.65 (2.44-8.85, p<0.001)
Tumor size (cm)		1.34 (1.24-1.45, p<0.001)
T	T1	
	T2	4.30 (1.84-10.08, p<0.001)
	T3	6.10 (2.59-14.36, p<0.001)
	T4	6.07 (3.02-12.21, p<0.001)
Vessel invasion	No	
	Yes	4.25 (2.90-6.23, p<0.001)
Hospitalization (d)		1.02 (1.00-1.05, p=0.029)
Absolute monocyte count (10^9/L)		2.72 (1.17-6.33, p=0.020)
HCT		0.98 (0.97-0.99, p<0.001)
LMR		0.92 (0.86-0.99, p=0.017)
PNI		0.98 (0.96-1.00, p=0.049)
Albumin (g/L)		0.96 (0.93-0.99, p=0.015)
A/G		0.43 (0.24-0.77, p=0.004)

LNM, lymph node metastasis; HCT, hematocrit; LMR, lymphocyte-monocyte ratio; PNI, prognostic nutritional index; A/G, albumin/globulin.

**Figure 2:**
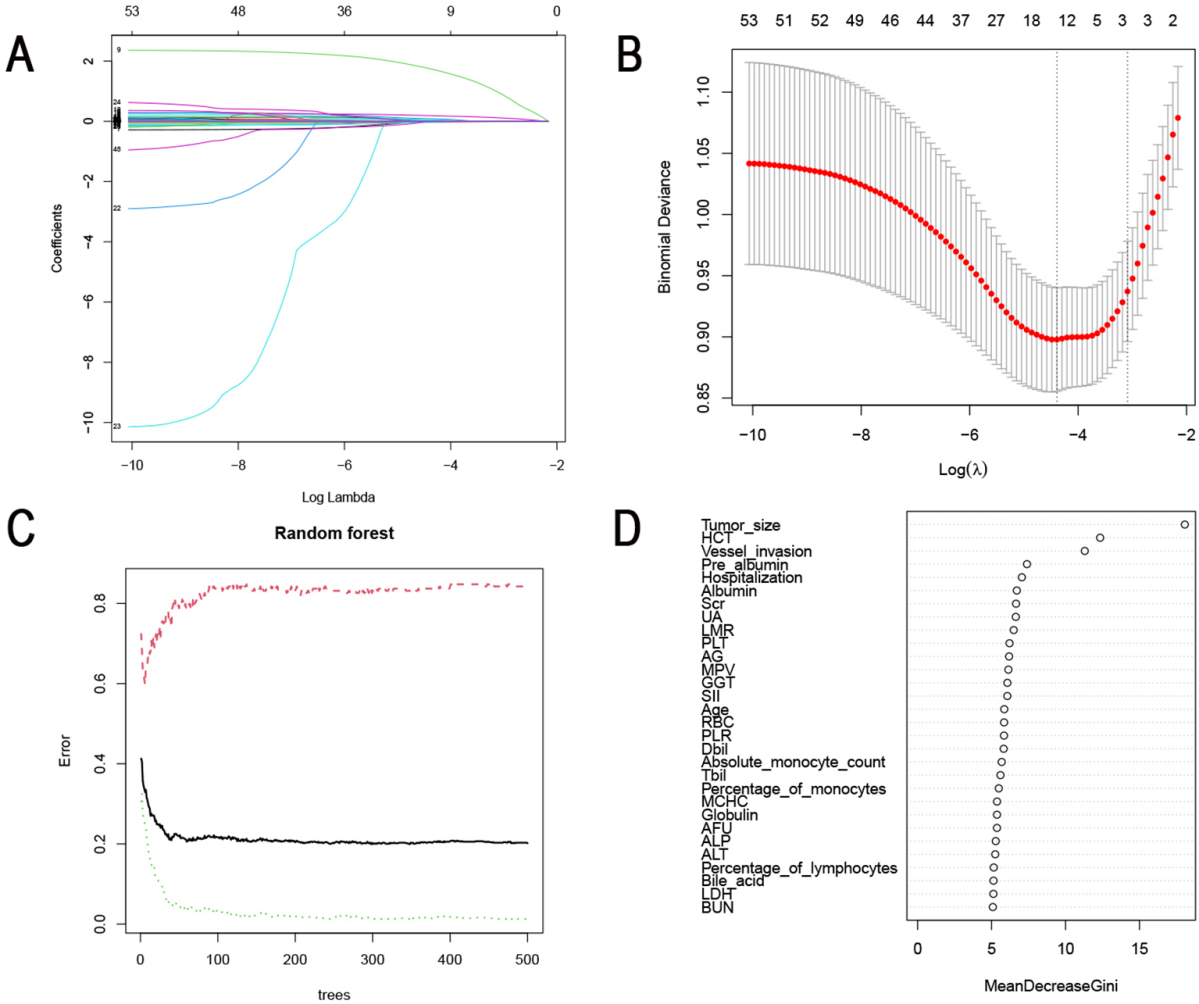
Identification of risk factors for LNM using machine learning algorithms. **(A)** Identification of the optimal penalization coefficient lambda (λ) in the LASSO model with 10-fold cross-validation in the training set. **(B)** LASSO coefficient profiles of 54 features. **(C)** The influence of the number of decision trees on the error rate. The x-axis represented the number of decision trees, and the y-axis indicated the error rate. **(D)** The importance of 29 features was ranked using RF. LNM, Lymph node metastasis; LASSO, Least absolute selection and shrinkage operator.

Subsequently, the LASSO algorithm was applied to screen for risk factors for LNM. As shown in **Figure 2A**, a total of 54 clinical parameters were integrated into the LASSO model, which can effectively penalize non-essential features. After ten-fold cross-validation, thirteen variables emerged as significant correlates under minimum criteria (**Figure 2B**), encompassing gender, tumor location, Borrmann type, differentiation type, maximum tumor diameter, vascular invasion, percentage of monocytes, percentage of eosinophils, hemoglobin (Hb), HCT, LMR, γ Glutamyl transpeptidase (GGT), α-L-fucosidase (AFU).

The RF machine learning algorithm further refined risk factor selection. The mean error rate was calculated separately for the node-positive and no-node-positive groups. The number of cross-validation error minimum when the tree is 299 (**Figure 2C**). We then scored the importance of clinical characteristics and visualized the ranking of them in **Figure 2D**. 29 clinically relevant features with scores exceeding 5 were considered risk factors for LNM. These included maximum tumor diameter, HCT, vascular invasion, pre-albumin, length of hospital stay, albumin, serum creatinine (Scr), uric acid (UA), LMR, PLT, A/G, mean platelet volume (MPV), GGT, SII, age, red blood cell count (RBC), PLR, direct bilirubin (Dbil), percentage of monocytes, absolute monocyte count, total bilirubin (Tbil), mean corpuscular hemoglobin concentration (MCHC), globulin, AFU, alkaline phosphatase (ALP), alanine aminotransferase (ALT), percentage of lymphocytes, bile acid, lactate dehydrogenase (LDH), and urea.

**Figure 3:**
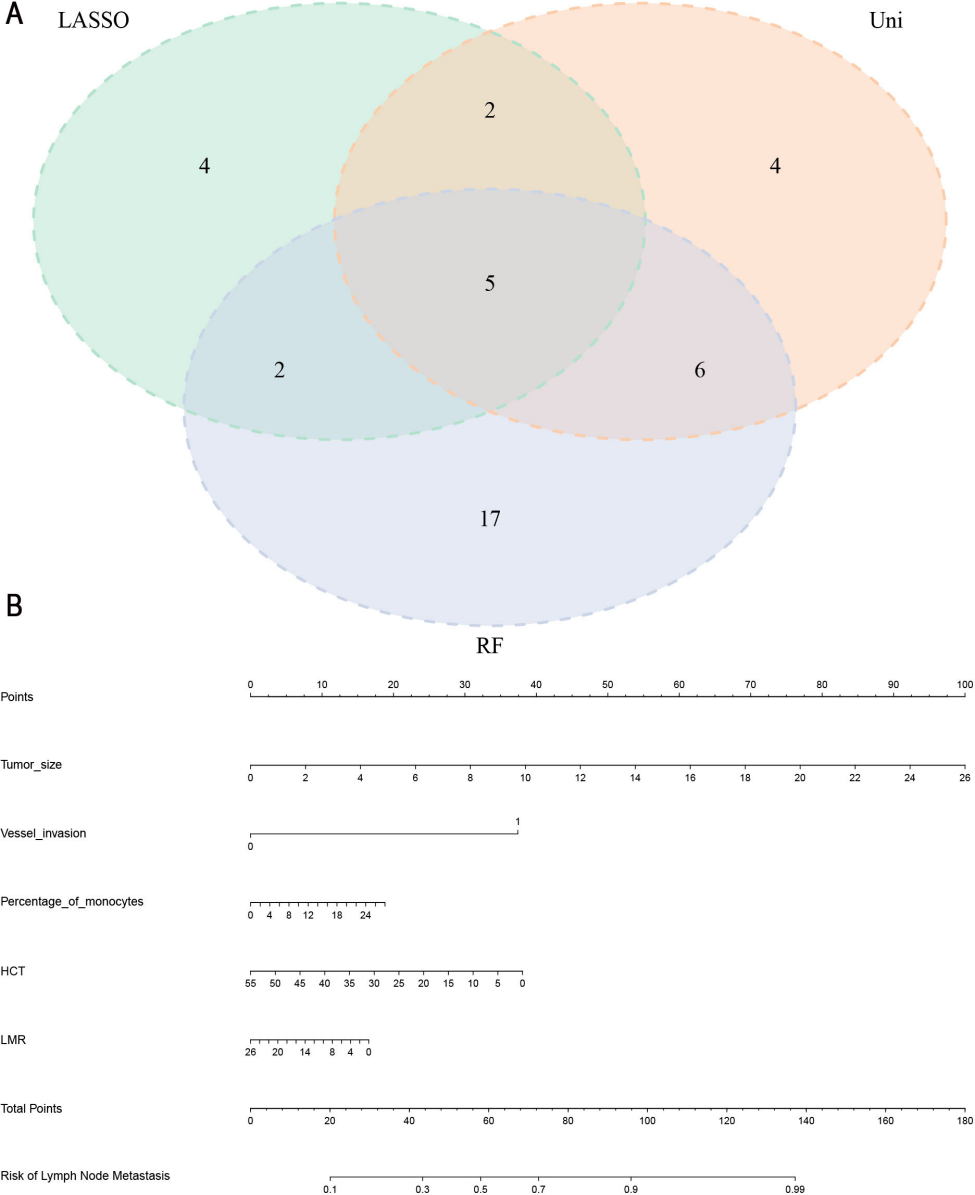
**(A)** Five common risk factors for LNM were visualized using a Venn diagram. LASSO, Least absolute selection and shrinkage operator; RF, random forest; Uni, univariate logistic regression analysis. **(B)** Nomogram for the prediction of LNM in gastric cancer. LNM, Lymph node metastasis; HCT, hematocrit; LMR, lymphocyte-monocyte ratio.

Harmonizing the results from univariate logistic regression, LASSO, and RF analyses, a comprehensive set of common LNM risk factors emerged. Visualization via the Venn diagram (**Figure 3A**) underscored the intersection of these factors, ultimately revealing maximum tumor diameter, vascular invasion, percentage of monocytes, HCT and LMR as the five pivotal variables for inclusion in subsequent nomogram analysis.

### Establishment and validation of the nomogram model

**Figure 4:**
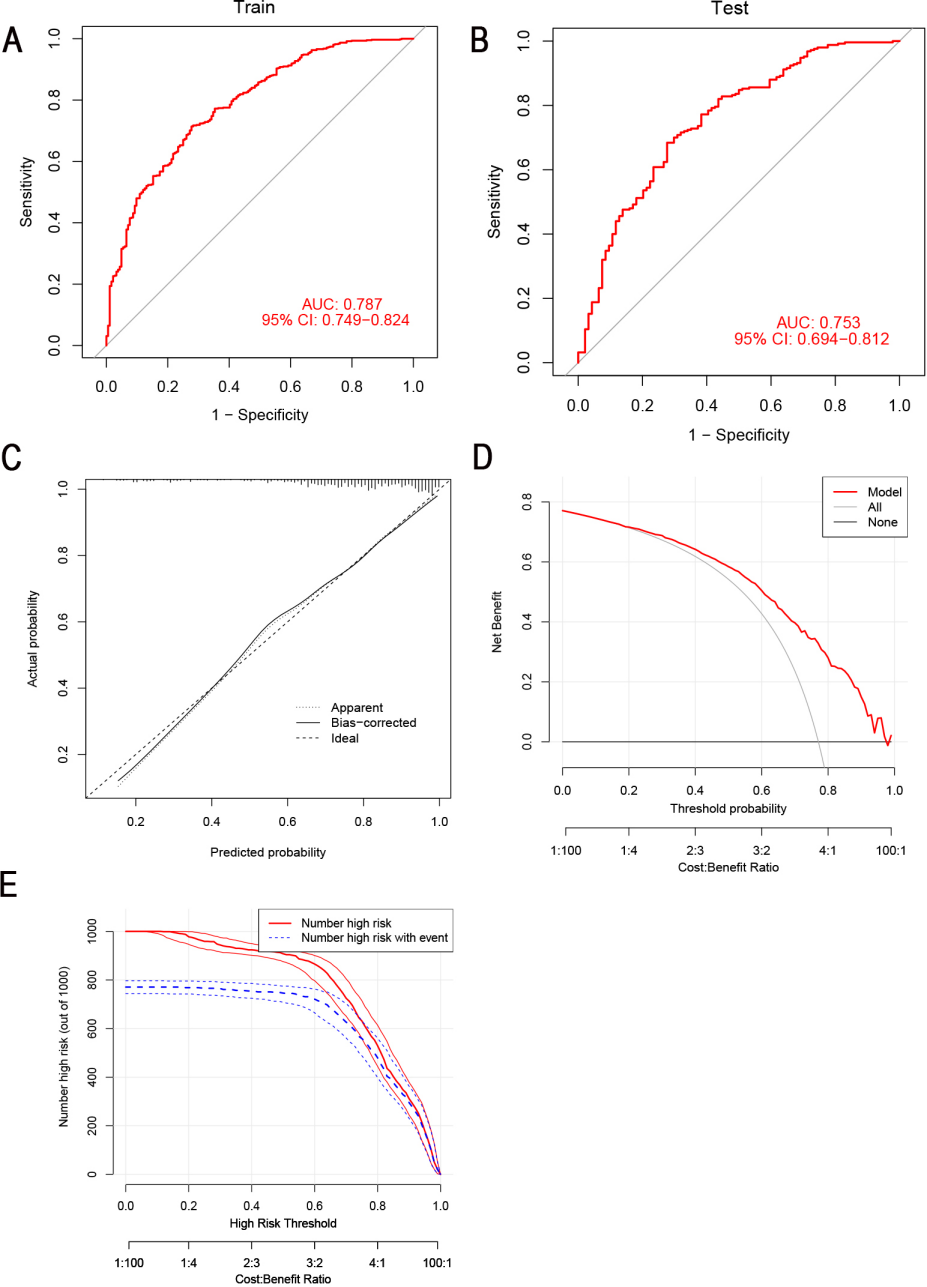
The predictive ability of the nomogram was verified. **(A, B)** ROC curves for the prediction of LNM in the training set and validation set. **(C)** Calibration curves in the training set. The x-axis represented the predicted probability from the nomogram, and the y-axis indicated the actual probability of LNM in gastric cancer patients. **(D)** DCA in the training set. The y-axis represented net benefits, calculated by subtracting the relative harm (false positives) from the benefits (true positives). The x-axis indicated the threshold probability. **(E)** Clinical impact curves of nomogram. The y-axis represented the number of people with high risk. The x-axis indicated the threshold probability. The red lines represented the number of individuals identified as high risk (LNM) by the model at the corresponding probability threshold. The blue lines represented the number of individuals who, at that same probability threshold, were classified by the model as high risk and actually experienced an outcome event (LNM). ROC, receiver operating characteristic curve; LNM, lymph node metastasis; DCA, decision curve analysis.

In the training set, a nomogram was crafted based on maximum tumor diameter, vascular invasion, percentage of monocytes, HCT and LMR. Each risk factor received a corresponding score, with the cumulative total score used to compute the probability of lymph node metastasis, as visually depicted in **Figure 3B**. The optimal cut-off value derived from the training set was determined as 0.758 with an AUC of 0.787 (95% CI: 0.749- 0.824), sensitivity (0.714), and specificity (0.723). In the validation set, the nomogram’s optimal cut-off value was established at 0.956, resulting in an AUC of 0.753 (95% CI: 0.694- 0.812). Sensitivity and specificity values were noted as 0.684 and 0.723, respectively. These metrics collectively affirmed the nomogram’s robust predictive capabilities in both the training and validation sets (**Figures 4A, B**). We employed a calibration curve to thoroughly evaluate the performance of the nomogram. The nomogram model underwent internal validation using the Bootstrap repeated self-sampling technique for 1000 iterations. The findings indicated that there was a minimal discrepancy of 0.011 between the simulated and actual curves in terms of absolute error. Calibration curves elucidated an excellent alignment between the nomogram’s predictions of LNM and the actual occurrences, as depicted in **Figures 4C**. DCA curves and clinical impact curves (**Figures 4D, E**) showed that the nomogram we built up had good clinical benefits. This concordance underscored the reliability and accuracy of the nomogram. Based on the **Supplementary Figure 1**, the validation cohort of calibration curves, DCA curves, and clinical decision curves showed similar results to those of the training cohort, indicating that the model has good predictive ability.

### Comparison of the LNM prediction model with others

**Figure 5:**
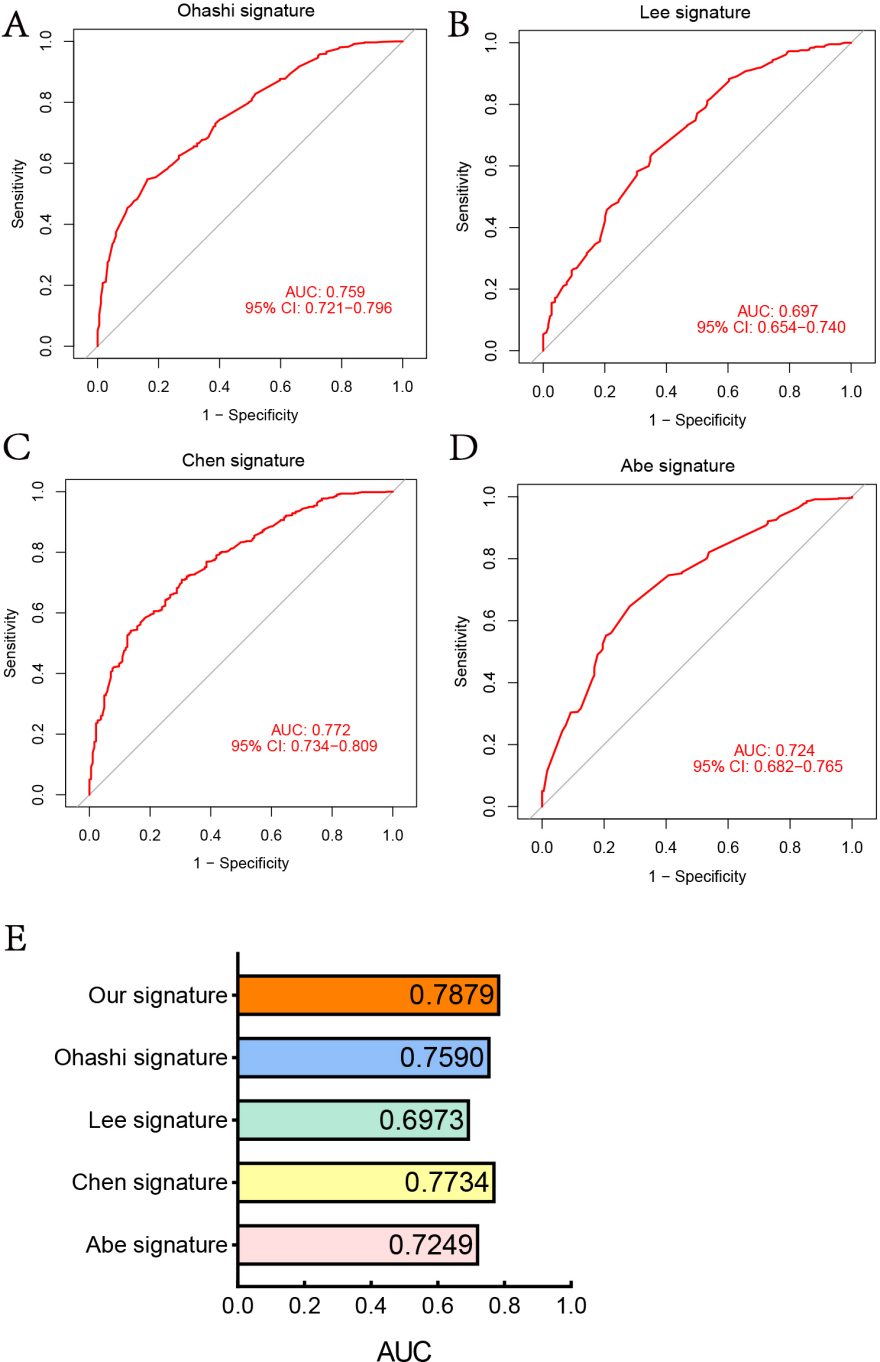
Comparison of the LNM prediction model with others. **(A)** ROC curve of 3-clinical-signature (Ohashi). **(B)** ROC curve of 3-clinical-signature (Lee). **(C)** ROC curve of 5-clinical-signature (Ohashi). **(D)** ROC curve of 4-clinical-signature (Abe). **(E)** AUC of five LNM prediction models.

We reviewed previously published studies on the prediction of LNM risk and selected three (20, 21), four (22), and five (23) clinical signature models for comparison with our LNM model. To ensure comparability among the models, we used the same method to construct nomogram models for each models and calculated the AUC values. As shown in **Figures 5A–E**, the AUC values for the four other models are lower than that of our LNM model.

### Evaluation of the importance of variables

**Figure 6:**
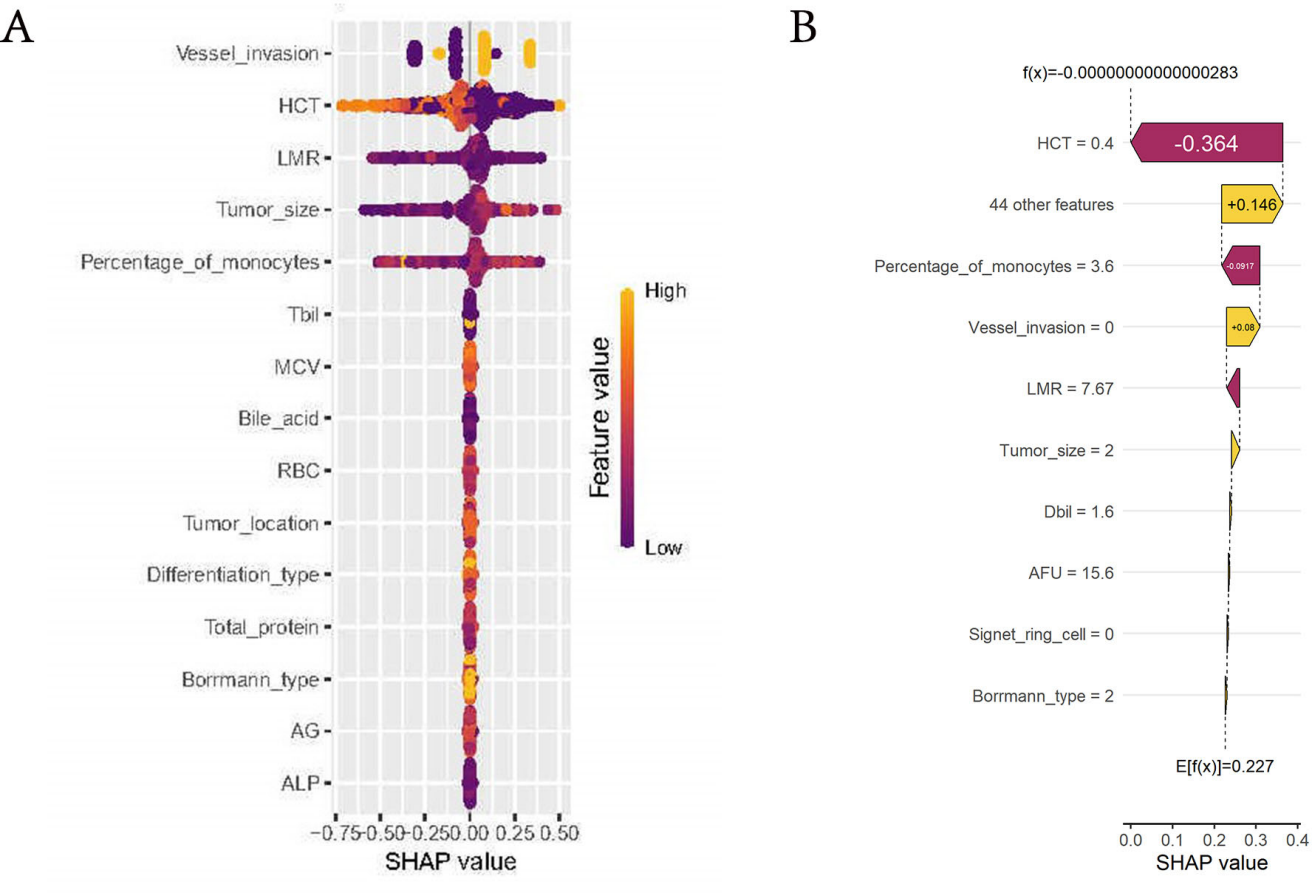
Evaluation of the importance of variables. **(A)** Beeswarm plot of the model. Generate SHAP values for each variable and reveal its relationship with LNM. The vertical axis represented the line where the SHAP value was 0. Variables on the right side of this line were yellow, indicating a positive contribution to the prediction of LNM, while variables on the right side of the line are purple, indicating a negative contribution. **(B)** Waterfall plot of the model. The horizontal axis at the bottom represented the SHAP values, indicating the impact of each feature on the prediction. Features to the right of the vertical dashed line contribute positively to the prediction of LNM, while those to the left contribute negatively.

We employed the SHAP algorithm to assess the significance of variables selected by various machine learning algorithms for our model. The Beeswarm plot and waterfall plot (**Figures 6A, B**) illustrated that variables were ranked in descending order based on their contribution to the model. This indicated that the most critical factors for LNM were, in order, vascular invasion, HCT, LMR, tumor size, and percentage of monocytes. Notably, the largest tumor diameter, vascular invasion and percentage of monocytes were positively correlated with LNM, while HCT and LMR were negatively correlated.

## Discussion

LNM stands as a pivotal determinant influencing the prognosis and comprehensive treatment decisions in gastric cancer patients. A retrospective study conducted by Kazuki Kano et al. at a singular medical center revealed that individuals with postoperative pathological stage II/III gastric cancer, marked by a heightened incidence of lymph node metastasis, exhibited diminished 5-year postoperative recurrence-free survival (RFS) and overall survival (OS) (24). In a separate investigation, Jun Eul Hwang et al. demonstrated that the quantity of metastatic lymph nodes in gastric cancer patients serves as a valuable guide for tailoring adjuvant chemotherapy decisions, particularly following D2 gastrectomy, with a pronounced impact on stage III gastric cancer patients (25).

Presently, the assessment of regional LNM in gastric cancer often relies on auxiliary examinations such as abdominal CT, nuclear medicine techniques (including positron emission tomography and single photon emission computed tomography), and endoscopic ultrasound. Abdominal CT offers valuable insights into lymph node characteristics, aiding in the determination of metastasis presence based on size and morphology (26, 27). However, these modalities may lack sensitivity in detecting subtle metastatic lesions. Nuclear medicine examinations, like PET-CT, utilize radioactive tracers to gauge glucose metabolism levels in lymph nodes (28), yet factors such as *H.pylori* infection, gastritis, and gastric peristalsis can influence detection accuracy (29–32). Endoscopic ultrasonography can provide insights into gastric cancer infiltration and adjacent lymph nodes, its invasiveness and cost limit its routine use in gastric cancer patients.

In this study, we have successfully integrated the preoperative blood test data of patients during their hospital stay with the postoperative tumor-specific data, enabling us to construct a comprehensive model. These markers were subjected to various machine learning methods to filter the clinical characteristics of gastric cancer patients. Ultimately, five key risk factors for LNM were identified: maximum tumor diameter, vascular invasion, percentage of monocytes, HCT and LMR. A nomogram model, employing these five indicators, was constructed and demonstrated robust predictive performance in both the training and validation sets. We further explained the extent to which the five variables contributed to LNM using the shap algorithm.

An expanding body of evidence underscores the intricate connection between chronic inflammatory states and cancer, with active involvement across various stages of tumorigenesis, proliferation, and metastasis (33–35). Various ratios of peripheral blood cells serve as bridges connecting the tumor microenvironment and systemic inflammatory factors. Key indicators such as LMR, NLR, PLR, and SII, derived from routine blood tests, offer a dynamic reflection of the delicate equilibrium between the immune system’s anti-tumor and pro-tumor functions. These indicators have demonstrated significant associations with cancer prognosis and LNM (36, 37).In this research, we observed a significant difference in PLR, LMR, and SII between the LNM and non-LNM groups, while NLR did not exhibit statistical disparities. Univariate logistic regression revealed that decreased LMR emerged as a risk factor for LNM in gastric cancer patients (OR=0.92, p=0.017). Notably, two machine learning methods also identified LMR as a significant risk factor for LNM. Therefore, our focus shifted to LMR in further investigations.

In a research involving 440,000 individuals, the level of LMR was found to have an inverse correlation with the risk of various cancers such as colorectal, gastric, renal, and ovarian cancers (38). Meta-analyses have underscored the significant association between a decreased LMR and diminished overall survival (OS) rates in patients with gastric cancer, while no discernible impact has been observed on disease-free survival (DFS) and recurrence-free survival (RFS). Low LMR is frequently associated with advanced age, LNM, distant metastasis, and elevated levels of carcinoembryonic antigen (CEA) (39).The intricate mechanisms underlying this association involve the interaction between lymphocytes, monocytes, and tumor cells. Lymphocytes play a crucial role in eliminating tumor cells by maintaining immune surveillance and detecting abnormalities (40). They can be categorized into T cells, B cells and NK cells (41). T cells exhibit anti-tumor effects and actively participate in cell-mediated immune responses against cancer (42). CD4+T cells assist in activating CD8+T cells which leads to apoptosis of cancerous cells (43). B lymphocytes possess the ability to produce antibodies and release cytokines, such as IL-6, INF-γ, and TNF-a. These cytokines are crucial in promoting the development of effector and memory T cells while indirectly modulating cellular immunity (44). It is worth noting that gastric cancer patients who have a high infiltration of CD20+B cells and CD8+T cells experience significantly prolonged overall survival (45, 46). However, when malnutrition, immune dysregulation, and inflammatory processes coexist, they collectively contribute to a decrease in lymphocyte count that compromises the immune response against tumors. In the tumor microenvironment, macrophages or monocytes known as tumor-associated macrophages (TAMs) play a crucial role in the pathogenesis of gastric cancer (47). Specifically, M2-type macrophages are responsible for promoting angiogenesis and extracellular matrix degradation while modulating the immune microenvironment and facilitating migration and progression of tumor cells (41, 48). Moreover, heightened TAM infiltration levels can confer resistance to chemotherapeutic drug like 5-fluorouracil in gastric cancer cells by activating reactive oxygen species and hypoxia-inducible factor 1α signaling pathways (48). The upregulation of peripheral monocytes may indicate an increased burden of TAMs within the tumor microenvironment.

The presence and progression of gastric cancer often coincide with the occurrence of anemia. Anemia can be attributed to two underlying factors: firstly, the tumor infiltrating blood vessels leading to hemorrhage; secondly, the tumor’s proliferative nature enhances iron absorption while reducing iron output (49). Clinical indicators for anemia include levels of Hb, HCT, MCV, MCH. In our investigation, we observed a significant decrease in all indicators of anemia within the cohort with LNM. Univariate logistic regression analysis revealed that only HCT emerged as a risk factor for LNM and was subsequently used to develop a nomogram model, highlighting the distinctive significance of HCT. HCT represents the proportion of red blood cells relative to blood volume, and previous retrospective studies have demonstrated its superiority over Hb in predicting OS among lung, breast, and gastric cancers (50, 51). This can be attributed to the fact that HCT is derived from fully functional red blood cells, providing a more accurate reflection of erythropoiesis capacity and oxygen-carrying capability.

Prior studies have consistently demonstrated the implication of vascular invasion in the lymph node and distant metastasis of gastric cancer, correlating with unfavorable prognostic outcomes (52). Our study echoes the significance of vascular invasion in the context of LNM. Vascular Endothelial Growth Factor (VEGF), a dimeric glycoprotein intimately linked to angiogenesis, is frequently found to be overexpressed in gastric cancer (53). Notably, the protein product of P53, a ubiquitous tumor suppressor gene, exerts inhibitory effects on VEGF expression, thereby suppressing angiogenesis (54). Within the realm of oncology, antiangiogenic agents, including monoclonal antibodies targeting VEGF, have gained widespread utilization (55).

Numerous prediction models for LNM in gastric cancer have been devised, but the majority have concentrated on early gastric cancer (EGC). For instance, Bang Wool Eom et al. crafted a model for EGC incorporating α1 catenin, CD44v6 biomarkers, and diverse clinicopathological parameters (AUC 0.83, 95% CI 0.766-0.895) (56). Fenglin Cai et al. devised a risk model for EGC, utilizing tumor size, depth of invasion, histological type, and lymphatic vascular involvement as key factors (57). However, considering the prevalence of advanced stage gastric cancer among patients, our research has redirected its attention towards stage II-III gastric cancer. Precise preoperative assessment of LNM risk is pivotal for optimal treatment strategy selection, particularly with the increasing advocacy for neoadjuvant therapy prior to surgery in cases of lymph-node metastasis (58, 59). Regrettably, models tailored for predicting LNM in stage II-III gastric cancer remain scarce. Xue Zhen et al. employed a neural network algorithm, encompassing indicators such as PLR, SII, tumor size, cN stage, Carcinoembryonic Antigen (CEA), and Cancer Antigen 199 (CA199) for stage II-III gastric cancer patients. Their model exhibited AUC values of 0.748 (95% CI: 0.717-0.776) in the training group and 0.717 (95% CI: 0.668-0.763) in the validation group (60). In contrast, our approach employed various machine learning methods to scrutinize variables, ultimately incorporating maximum tumor diameter, vascular invasion, percentage of monocytes, HCT and LMR as the pivotal risk factors. The visualization of this regression model through a nomogram generates individual probabilities of LNM events and enhances its clinical utility.

While this study contributes valuable insights, it is essential to acknowledge certain limitations. Primarily, being a single-center retrospective study, the patient cohort was exclusively composed of individuals from the East Asian population, introducing a potential regional bias. To enhance the clinical generalizability of our findings, this study did not incorporate abdominal CT, PET-CT, and other imaging modalities. Future research endeavors could explore the development of collaborative image-based models, thereby augmenting the model’s applicability across diverse populations.

## Conclusion

In summary, by utilizing machine learning algorithms, we created a nomogram to accurately predict the risk of lymph node metastasis in stage II-III gastric cancer. This provides a useful tool for personalized risk assessment in clinical decision-making.

## Data Availability

The original contributions presented in the study are included in the article/**Supplementary Material**, further inquiries can be directed to the corresponding author/s.
