# Supplementary material for: Construction and validation of a nomogram model for lymph node metastasis of stage II-III gastric cancer based on machine learning algorithms
Source: Front Oncol. 2024 Oct 8;14:1399970. doi: 10.3389/fonc.2024.1399970 (PMC11493538; doi:10.3389/fonc.2024.1399970)
Supplement: Supplementary file 2 [file Image1.pdf]

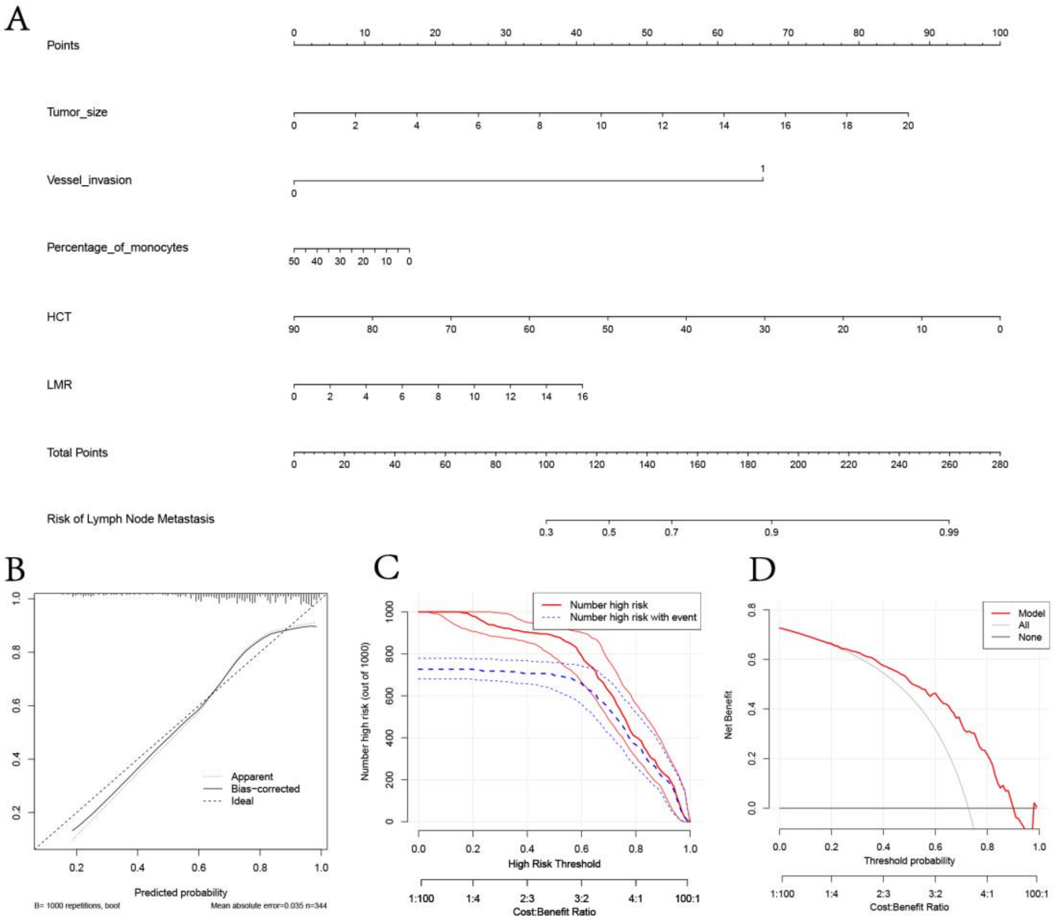

Supplementary Figure 1. (A) Nomogram for the prediction of LNM in gastric cancer in the validation cohort. (B) Calibration curves in the validation cohort. The x-axis represented the predicted probability from the nomogram, and the y-axis indicated the actual probability of LNM in gastric cancer patients. (C) Clinical impact curves of nomogram in the validation cohort. The y-axis represented the number of people with high risk. The x-axis indicated the threshold probability. The red lines represented the number of individuals identified as high risk (LNM) by the model at the corresponding probability threshold. The blue lines represented the number of individuals who, at that same probability threshold, were classified by the model as high risk and actually experienced an outcome event (LNM). (D) DCA in the validation cohort. The y-axis represented net benefits, calculated by subtracting the relative harm (false positives) from the benefits (true positives). The x-axis indicated the threshold probability. LNM, Lymph node metastasis; HCT, hematocrit; LMR, lymphocyte-monocyte ratio; DCA, decision curve analysis.
